# Supplementary material for: Manipulating network connectance by altering plant attractiveness
Source: PeerJ. 2023 Nov 9;11:e16319. doi: 10.7717/peerj.16319 (PMC10640842; doi:10.7717/peerj.16319)
Supplement: Supplemental Information 1 [file peerj-11-16319-s001.docx]

Supplemental material for: **Manipulating network connectance by altering plant attractiveness**

**Supplemental methods:**

*Seeding and planting the plots*

We seeded each plot with 4.5 g (~608 seeds/m^2^) of the non-native *Phacelia tanacetifolia*, which is commonly recommended in pollinator plantings in Europe (Rundlöf, Lundin & Bommarco, 2018). Then we transplanted four individuals of each of the native perennial species: *Plantago lanceolata, Hypochaeris radicata, Filipendula ulmaria*, and *Epilobium hirsutum*, two individuals of *Cirsium vulgare*, and one individual of *Origanum vulgare* into each plot. Within a species, all the perennials were sourced from the same populations and were approximately the same size upon planting. *P. lanceolata, C. vulgare, F. ulmaria, E. hirsutum*, and *H. radicata* individuals were sourced from populations in a wildlife conservation area in Kilkenny, Ireland, while *O. vulgare* individuals were sourced from a commercial supplier. After planting, the plants were watered for a month before treatments were initiated so the plants could establish. Throughout the season, plots were watered regularly and non-target plants (other than the species/individuals we planted) were removed by hand as needed. We also prevented *P. tanacetifolia* from competing with the perennial individuals with hand weeding; densities of this species were variable in the different plots, but we removed individuals when they overgrew the perennials.

*Detailed site descriptions*

All sites were planted within the urban Dublin matrix, with mixed grassy and treed areas and impermeable surfaces (Fig. S6). The soil substrate of all sites was “made ground” (Geological Survey Ireland, Department of Communications, Climate Action & Environment, using the Teagasc soils layer, Fig. S1). A soil analysis conducted in 2017 found no interaction between our experimental treatments and the soil composition of the first four sites (Russo et al, 2020).

Four sites were established in 2017: GNI, RTE, UCDR, and TCD. GNI and RTE were located on private property owned by local businesses which agreed to let us use their land for the experiment, but had no vested interest in the outcome or competing financial interests. UCDR and TCD were university owned properties (University College, Dublin and Trinity College Dublin, respectively). At all four sites, plots were located in areas that were regularly mown, but the surrounds varied from open grassy areas (GNI), urban areas (RTE), an apple orchard (UCDR), and a botanical garden (TCD).

Four sites were established in 2018: MI, RV, AE, and UCDM. MI, RV, and AE were located on private property owned by local businesses which agreed to let us use their land for the experiment, but had no vested interest in the outcome or competing financial interests. AE was a managed tourist farm and RV was a primary school ground, while MI and UCDM were private and public university properties, respectively. Three of four sites were regularly mown, while UCDM was not mown. The surrounds varied from open grassy areas (MI, RV), to a property managed for tourists with a hobby farm (AE), to the unmanaged and weedy UCDM site. *Agrochemical treatments*

**Fertilizer**

For a replicable fertilizer treatment for our experiment, we used measured levels of nitrates and phosphorus in Irish groundwater (Craig & Mannix, 2009). The Irish EPA assessment did not address groundwater potassium, so we used run-off levels measured in Bertol et. al (2007) for conventional agricultural systems. We other studies used to confirm relatively common run-off values for nitrogen, phosphorus, and potassium (Korsaeth & Eltun, 2000; Bertol et al., 2007). To achieve the target concentrations, we purchased each element (NPK) separately and measured them out by weight using the water solubility percentage listed on the package. These were mixed, then diluted in 10 l of water for application purposes. For nitrogen (N), we purchased sulphate of ammonia ((NH4)2SO4, ®Westland), which had 21% water soluble ammoniacal nitrogen. For the phosphorus (P), we used superphosphate (Ca(H2PO4)2, ®Westland), which had 7% water soluble P. For the potassium (K), we used sulphate of potash (K2SO4, ®Westland), with 48% water soluble K.

**Glyphosate**

Glyphosate is not mobile in the groundwater system and is rarely detected in high concentrations (Saunders & Pezeshki, 2015), but run-off levels can be very high (up to 7.6 mg/l, (Jones, Hammond & Relyea, 2010)) immediately after application, especially if it is sprayed over water (Edwards, Triplett & Kramer, 1980). Large groundwater surveys rarely detect the 0.7 mg/l maximum contaminant level (MCL) set by the US EPA (2003). However, plants adjacent to crop fields are at the highest risk of exposure to glyphosate run-off, which has been shown to have detrimental effects on these plants (Saunders & Pezeshki, 2015). Furthermore, phosphorus fertilization can increase the run-off of glyphosate (Sasal et al., 2015). We used 0.7 mg/l MCL (US EPA 2003) as the maximum concentration applied and decreased this concentration across the summer. We purchased “®Roundup” concentrate from a hardware store, and then measured out a volume using the percentage of active ingredient listed on the package, which we diluted in 10 l of water before application.

**Supplemental References**

Bertol I, Engel FL, Mafra AL, Bertol OJ, Ritter SR. 2007. Phosphorus, potassium and organic carbon concentrations in runoff water and sediments under different soil tillage systems during soybean growth. *Soil and Tillage Research* 94:142–150. DOI: 10.1016/J.STILL.2006.07.008.

Craig M, Mannix A. 2009. Groundwater quality. McGarrigle, M., Lucey, L., Ó Cinnéide, M.(Eds.), *Water Quality in Ireland*, 42.

Edwards WM, Triplett GB, Kramer RM. 1980. A watershed study of glyphosate transport in runoff. *Journal of Environment Quality* 9:661. DOI: 10.2134/jeq1980.00472425000900040024x.

Jones DK, Hammond JI, Relyea RA. 2010. Roundup® and amphibians: The importance of concentration, application time, and stratification. *Environmental Toxicology and Chemistry* 29:446–454. DOI: 10.1002/etc.240.

Korsaeth A, Eltun R. 2000. Nitrogen mass balances in conventional, integrated and ecological cropping systems and the relationship between balance calculations and nitrogen runoff in an 8-year field experiment in Norway. *Agriculture, Ecosystems & Environment* 79:199–214. DOI: 10.1016/S0167-8809(00)00129-8.

Rundlöf M, Lundin O, Bommarco R. 2018. Annual flower strips support pollinators and potentially enhance red clover seed yield. Ecology and Evolution 8:7974–7985. DOI: 10.1002/ece3.4330.

Sasal MC, Demonte L, Cislaghi A, Gabioud EA, Oszust JD, Wilson MG, Michlig N, Beldomenico HR, Repetti MR. 2015. Glyphosate loss by runoff and its relationship with Phosphorus fertilization. *Journal of Agricultural and Food Chemistry* 63:4444–4448.

Saunders L, Pezeshki R. 2015. Glyphosate in runoff waters and in the root-zone: a review. *Toxics* 3:462–480. DOI: 10.3390/toxics3040462.

**Table S1.** Species and morphospecies of flower-visiting insects collected during the experiment, sorted by order, family, and species/morphospecies.

| **Order** | **Family** | **Species/Morphospecies** | **Count** |
| --- | --- | --- | --- |
| Coleoptera | Cantharidae | *Rhagonycha fulva* | 5 |
| Diptera | Anthomyiidae | Anthomyiid morph1 | 9 |
|  |  | Anthomyiid morph2 | 17 |
|  | Calliphoridae | Calliphorid morph1 | 1 |
|  |  | *Lucilia richardsi* | 1 |
|  | Dolichopodidae | Dolichopodid morph1 | 2 |
|  | Empididae | Empid morph2 | 5 |
|  |  | *Empis livida* | 1 |
|  | Muscidae | Muscid morph1 | 1 |
|  |  | Muscid morph2 | 1 |
|  |  | Muscid morph3 | 1 |
|  |  | Muscid morph4 | 1 |
|  |  | Muscid morph6 | 1 |
|  | Syrphidae | *Cheilosia albitarsus* | 2 |
|  |  | *Chrysogaster solstitialis* | 1 |
|  |  | *Chrysotoxum bicinctum* | 1 |
|  |  | *Dasysyrphus albostriatus* | 3 |
|  |  | *Dasysyrphus tricinctus* | 1 |
|  |  | *Epistrophe grossulariae* | 1 |
|  |  | *Episyrphus balteatus* | 86 |
|  |  | *Eristalis horticola* | 1 |
|  |  | *Eristalis pertinax* | 6 |
|  |  | *Eristalis tenax* | 3 |
|  |  | *Eupeodes corollae* | 6 |
|  |  | *Eupeodes latifasciatus* | 11 |
|  |  | *Eupeodes luniger* | 12 |
|  |  | *Ferdinandea cuprea* | 1 |
|  |  | *Helophilus pendulus* | 3 |
|  |  | *Herinigia heringi* | 1 |
|  |  | *Melanostoma mellinum* | 6 |
|  |  | *Melanostoma scalare* | 23 |
|  |  | *Meliscaeva auricollis* | 11 |
|  |  | *Myathropa florea* | 8 |
|  |  | *Neoascia podagrica* | 11 |
|  |  | *Neocnemodon brevidens* | 1 |
|  |  | *Neocnemodon vitripennis* | 1 |
|  |  | *Platycheirus albimanus* | 102 |
|  |  | *Platycheirus angustatus* | 1 |
|  |  | *Platycheirus clypeatus* | 1 |
|  |  | *Platycheirus scutatus* | 47 |
|  |  | *Ripponesia splendens* | 1 |
|  |  | *Scaeva pyrastri* | 6 |
|  |  | *Sphaerophoria scripta* | 74 |
|  |  | *Syritta pipiens* | 17 |
|  |  | *Syrphus ribesii* | 50 |
|  |  | *Syrphus torvus* | 2 |
|  |  | *Syrphus vitripennis* | 15 |
|  | Tachinidae | *Siphona geniculata* | 28 |
|  | Tephritidae | *Terellia serratulae* | 2 |
|  | Ulidiidae | *Seioptera vibrans* | 1 |
|  | unknown | Diptera morph1 | 1 |
|  |  | Diptera morph2 | 1 |
|  |  | Diptera morph3 | 1 |
|  |  | Diptera morph4 | 1 |
| Hemiptera | Miridae | Mirid morph1 | 2 |
|  |  | *Orthotylus* morph1 | 2 |
|  |  | *Stenotus binotatus* | 2 |
| Hymenoptera | Andrenidae | *Andrena bicolor* | 5 |
|  |  | *Andrena minitula* | 1 |
|  |  | *Andrena subopaca* | 1 |
|  | Apidae | *Apis mellifera* | 243 |
|  |  | *Bombus cryptarum* | 1 |
|  |  | *Bombus hortorum* | 2 |
|  |  | *Bombus hypnorum* | 1 |
|  |  | *Bombus lapidarius* | 68 |
|  |  | *Bombus lucorum* agg. | 408 |
|  |  | *Bombus muscorum* | 21 |
|  |  | *Bombus pascuorum* | 243 |
|  |  | *Bombus pratorum* | 93 |
|  |  | *Bombus sylvestris* | 1 |
|  |  | *Bombus terrestris* | 92 |
|  | Braconidae | Braconid morph1 | 1 |
|  | Colletidae | *Hylaeus communis* | 47 |
|  |  | *Hylaeus confusus* | 3 |
|  | Crabronidae | *Ectemnius cavifrons* | 1 |
|  | Halictidae | *Halictus rubicundus* | 1 |
|  |  | *Lasioglossum albipes* | 7 |
|  |  | *Lasioglossum calceatum* | 7 |
|  |  | *Lasioglossum cupromicans* | 2 |
|  |  | *Lasioglossum fratellum* | 4 |
|  |  | *Lasioglossum leucopus* | 23 |
|  |  | *Lasioglossum villosulum* | 10 |
|  | Ichneumonidae | Cryptinae morph1 | 2 |
|  |  | Ichneumonid morph1 | 1 |
|  | Megachildae | *Megachile versicolor* | 6 |
|  |  | *Megachile willughbiella* | 1 |
|  | Vespidae | *Vespula vulgaris* | 1 |
| Lepidoptera | Nymphalidae | *Maniola jurtina* | 1 |
|  | Pieridae | *Anthocharis cardamines* | 1 |
|  |  | *Pieris brassicae* | 2 |
|  |  | *Pieris napi* | 3 |
|  |  | *Pieris rapae* | 1 |

**Table S2.** Species codes used in Figure S3.

| Species/Morphospecies | Code |  | Species/Morphospecies cont. | Code cont. |
| --- | --- | --- | --- | --- |
| *Andrena bicolor* | 1 |  | *Hylaeus communis* | 43 |
| *Andrena minitula* | 2 |  | *Hylaeus confusus* | 44 |
| *Andrena subopaca* | 3 |  | *Lasioglossum albipes* | 45 |
| *Anthocharis cardamines* | 4 |  | *Lasioglossum calceatum* | 46 |
| Anthomyiid morph1 | 5 |  | *Lasioglossum cupromicans* | 47 |
| Anthomyiid morph2 | 6 |  | *Lasioglossum fratellum* | 48 |
| *Apis mellifera* | 7 |  | *Lasioglossum leucopus* | 49 |
| *Bombus hortorum* | 8 |  | *Lasioglossum villosulum* | 50 |
| *Bombus hypnorum* | 9 |  | *Lucilia richardsi* | 51 |
| *Bombus lapidarius* | 10 |  | *Maniola jurtina* | 52 |
| *Bombus lucorum* agg. | 11 |  | *Megachile versicolor* | 53 |
| *Bombus muscorum* | 12 |  | *Megachile willughbiella* | 54 |
| *Bombus pascuorum* | 13 |  | *Melanostoma mellinum* | 55 |
| *Bombus pratorum* | 14 |  | *Melanostoma scalare* | 56 |
| *Bombus sylvestris* | 15 |  | *Meliscaeva auricollis* | 57 |
| *Bombus terrestris* | 16 |  | Muscid morph1 | 58 |
| Calliphorid morph1 | 17 |  | Muscid morph2 | 59 |
| *Cheilosia albitarsus* | 18 |  | Muscid morph3 | 60 |
| *Chrysogaster solstitialis* | 19 |  | Muscid morph4 | 61 |
| *Chrysotoxum bicinctum* | 20 |  | Muscid morph6 | 62 |
| *Dasysyrphus albostriatus* | 21 |  | *Myathropa florea* | 63 |
| *Dasysyrphus tricinctus* | 22 |  | *Neoascia podagrica* | 64 |
| Diptera morph1 | 23 |  | *Neocnemodon brevidens* | 65 |
| Diptera morph2 | 24 |  | *Neocnemodon vitripennis* | 66 |
| Diptera morph3 | 25 |  | *Pieris brassicae* | 67 |
| Diptera morph4 | 26 |  | *Pieris napi* | 68 |
| Dolichopodid morph1 | 27 |  | *Pieris rapae* | 69 |
| *Ectemnius cavifrons* | 28 |  | *Platycheirus albimanus* | 70 |
| Empid morph2 | 29 |  | *Platycheirus angustatus* | 71 |
| *Empis livida* | 30 |  | *Platycheirus clypeatus* | 72 |
| *Epistrophe grossulariae* | 31 |  | *Platycheirus scutatus* | 73 |
| *Episyrphus balteatus* | 32 |  | *Rhagonycha fulva* | 74 |
| *Eristalis horticola* | 33 |  | *Ripponesia splendens* | 75 |
| *Eristalis pertinax* | 34 |  | *Scaeva pyrastri* | 76 |
| *Eristalis tenax* | 35 |  | *Seioptera vibrans* | 77 |
| *Eupeodes corollae* | 36 |  | *Siphona geniculata* | 78 |
| *Eupeodes latifasciatus* | 37 |  | *Sphaerophoria scripta* | 79 |
| *Eupeodes luniger* | 38 |  | *Stenotus binotatus* | 80 |
| *Ferdinandea cuprea* | 39 |  | *Syritta pipiens* | 81 |
| *Halictus rubicundus* | 40 |  | *Syrphus ribesii* | 82 |
| *Helophilus pendulus* | 41 |  | *Syrphus torvus* | 83 |
| *Herinigia heringi* | 42 |  | *Syrphus vitripennis* | 84 |

**Table S3. See supplementary files.**

**Table S4.** Results of GLMMs using z-scores of the network measures relative to null models created using r2dtable in the package *bipartite*. This includes the model structure with response variable, random effect, number of observations, fixed effect, and contrasts. The results include the effect size, T value, P value, and marginal and conditional R^2^.

| **Response** | **Random Effect** | **Observations** | **Fixed Effect** | **Contrast** | **Estimate** | **T value** | **P value** | **R^2^m** | **R^2^c** |
| --- | --- | --- | --- | --- | --- | --- | --- | --- | --- |
| NODF z-scores | Block | 30 obs, 8 blocks | Trt | C-F | -0.66 | -1.02 | 0.31 | 0.28 | 0.38 |
|  |  |  |  | **C-H** | **1.62** | **2.41** | **0.02** |  |  |
|  |  |  |  | C-HF | 0.2 | 0.3 | 0.76 |  |  |
| NODFc z-scores | Block | 30 obs, 8 blocks | Trt | C-F | 0.46 | 0.62 | 0.53 | 0.11 | 0.13 |
|  |  |  |  | C-H | -0.11 | -0.15 | 0.88 |  |  |
|  |  |  |  | C-HF | -0.88 | -1.19 | 0.23 |  |  |
| Connectance z-scores | Block | 30 obs, 8 blocks | Trt | C-F | -0.66 | -1.02 | 0.31 | 0.28 | 0.38 |
|  |  |  |  | **C-H** | **1.62** | **2.41** | **0.02** |  |  |
|  |  |  |  | C-HF | 0.2 | 0.3 | 0.76 |  |  |
| Weighted degree z-score | Block | 30 obs, 8 blocks | Trt | C-F | 0.16 | 0.17 | 0.87 | 0.09 | 0.54 |
|  |  |  |  | C-H | 1.65 | 1.69 | 0.09 |  |  |
|  |  |  |  | C-HF | -0.4 | -0.42 | 0.67 |  |  |
| Unweighted degree z-scores | Block | 30 obs, 8 blocks | Trt | C-F | -0.05 | -0.08 | 0.94 | 0.17 | 0.53 |
|  |  |  |  | **C-H** | **1.79** | **2.41** | **0.02** |  |  |
|  |  |  |  | C-HF | -0.31 | -0.44 | 0.66 |  |  |
| Modularity z-scores | Block | 30 obs, 8 blocks | Trt | C-F | 1.39 | 1.12 | 0.26 | 0.13 | 0.37 |
|  |  |  |  | C-H | -0.42 | -0.4 | 0.69 |  |  |
|  |  |  |  | C-HF | 2.11 | 1.7 | 0.09 |  |  |

**Figure S1**


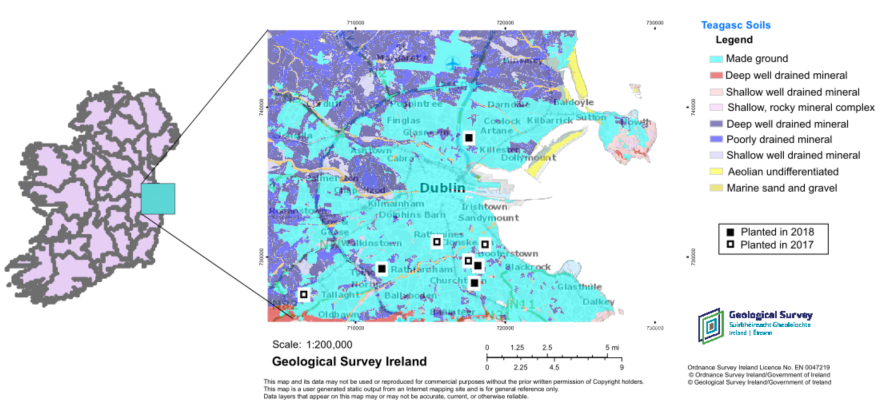


**Fig. S1**. Field sites in Dublin with background colors showing soil types, classified by Teagasc. Map downloaded (edited for clarity) from the Geological Survey Ireland, Department of Communications, Climate Action & Environment, using the Teagasc soils layer (https://dcenr.maps.arcgis.com/apps/MapSeries/index.html, accessed 19/06/2019). Figure contains Irish Public Sector Data (Geological Survey) licensed under a Creative Commons Attribution 4.0 International (CC BY 4.0) license. All research plots were located on made ground. Filled squares represent sites established in 2018, and open squares represent sites established in 2017. This figure is modified from Russo et al. (2020).

**Figure S2**

**
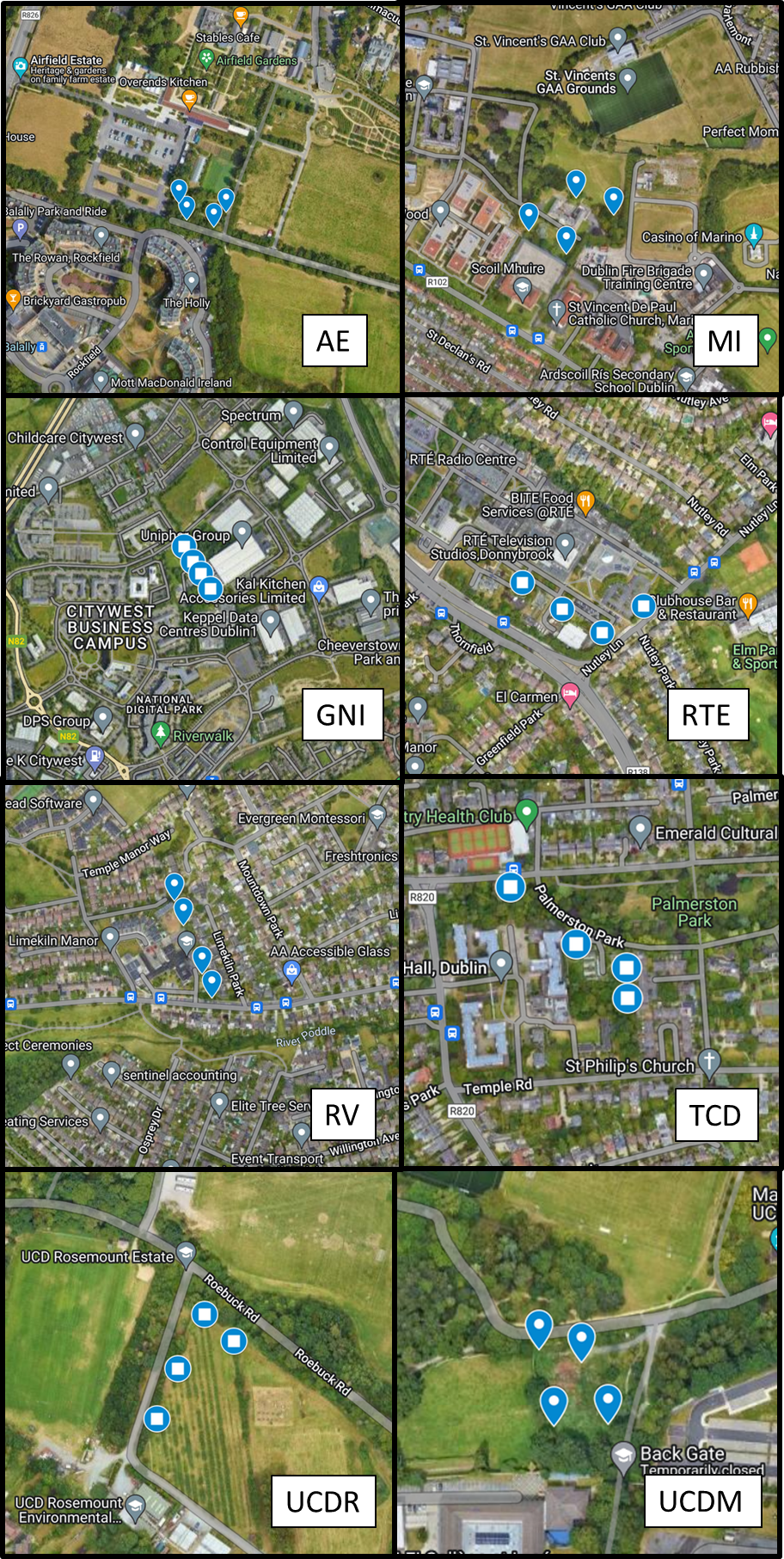
Fig. S2**. Aerial images (©Google MapData 2023) of the eight study sites, with plots indicated by blue markers. Plots established in 2017 are indicated by circles, while plots established in 2018 are marked by pins.

**Figure S3**


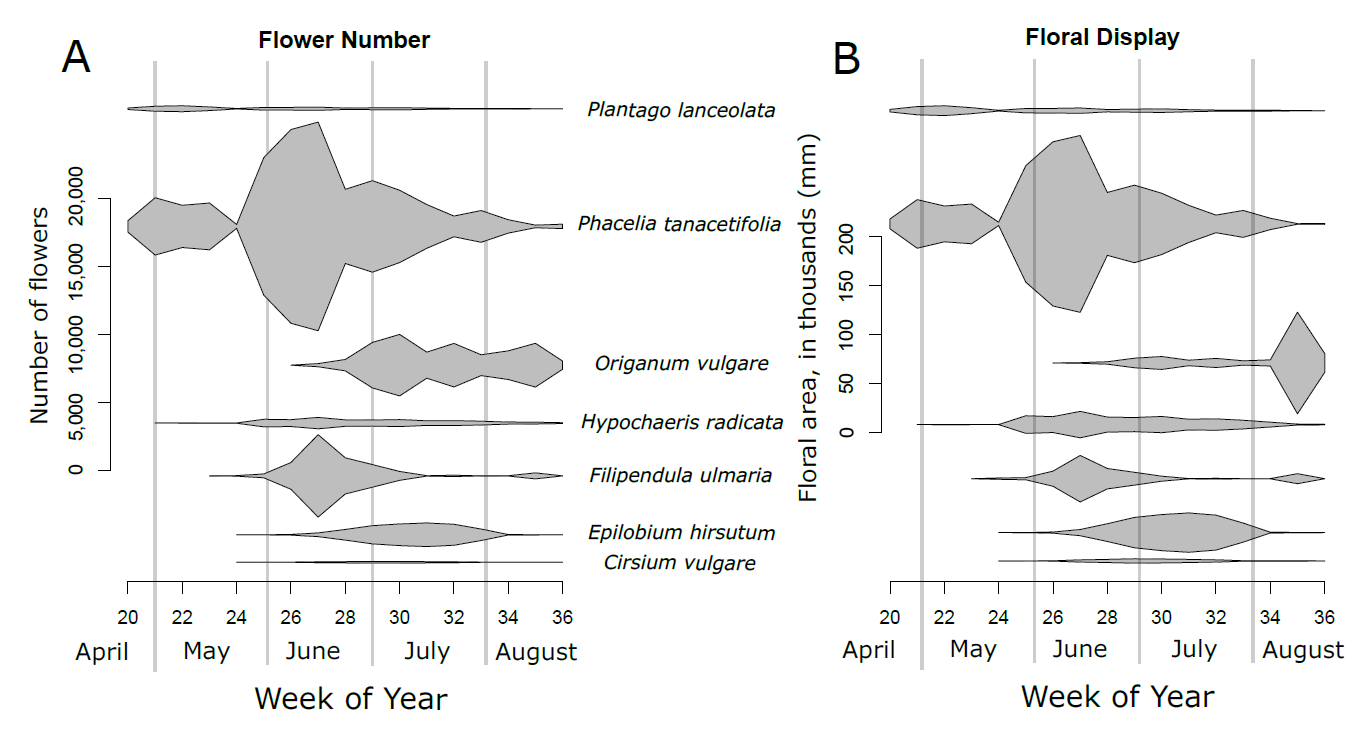


**Fig. S3.** Plot of the flowering phenology of the seven plant species included in the study, including number of flowers (A), and size of the floral display (B), calculated as the number of flowers multiplied by average flower size. The first two plant species began to bloom in late April and continued to bloom through August for both years. Bloom in the seven species overlapped for most (10 out of 16 weeks) of the field season.

**Figure S4**


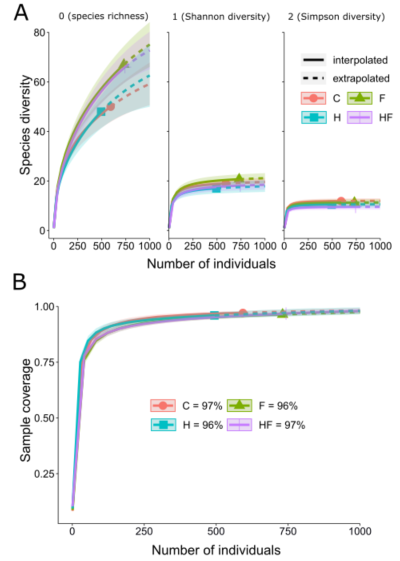


**Fig. S4.** A rarefaction analysis of the flower-visiting insects collected in this study. The top panel (A) shows the three Hill numbers among the four experimental treatments (C = control, F = fertilizer, H = herbicide, HF = combination). The shaded area around the lines indicates the 95% confidence interval of the expected diversity values of the four treatments. The bottom panel (B) shows the sample coverage for the four experimental treatments, including the percent of estimated diversity captured. In all treatments, we collect either 96 or 97% of the expected insect species richness.

**Figure S5**


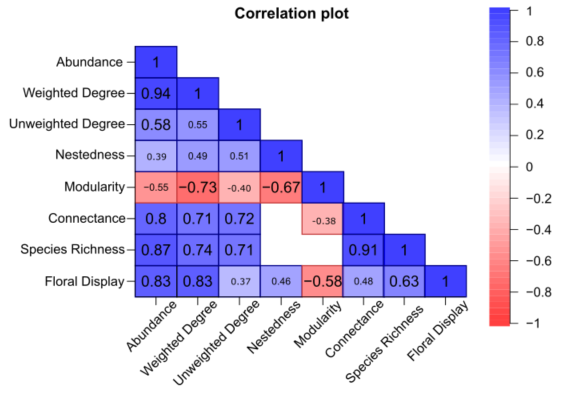


**Fig. S5.** Triangular correlation matrix between measured attributes of the networks. Numbers represent significant (P < 0.05) Pearson correlation coefficients. Larger font size indicates smaller P values. Negative correlations are indicated in red, while positive correlations are indicated in blue. Darker colors indicate correlation coefficients of greater magnitude. Associations in white with no numbers are non-significant (P > 0.05).

**Figure S6**


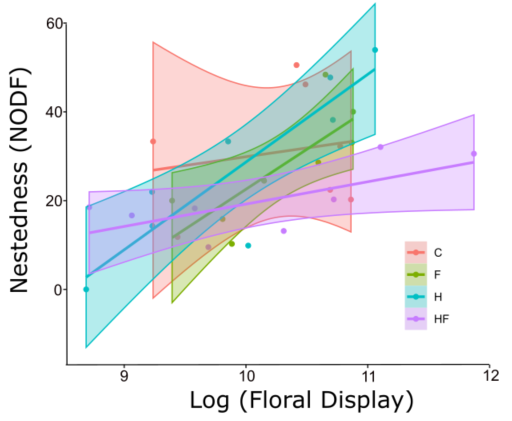


**Fig. S6.** Graph showing linear relationships between the log-transformed floral display size and network nestedness. The colors indicate experimental treatment: red = control, green = fertilizer, blue = herbicide, purple = combination. There is a significant interaction between the size of the floral display and experimental treatment for network nestedness.

**Figure S7**


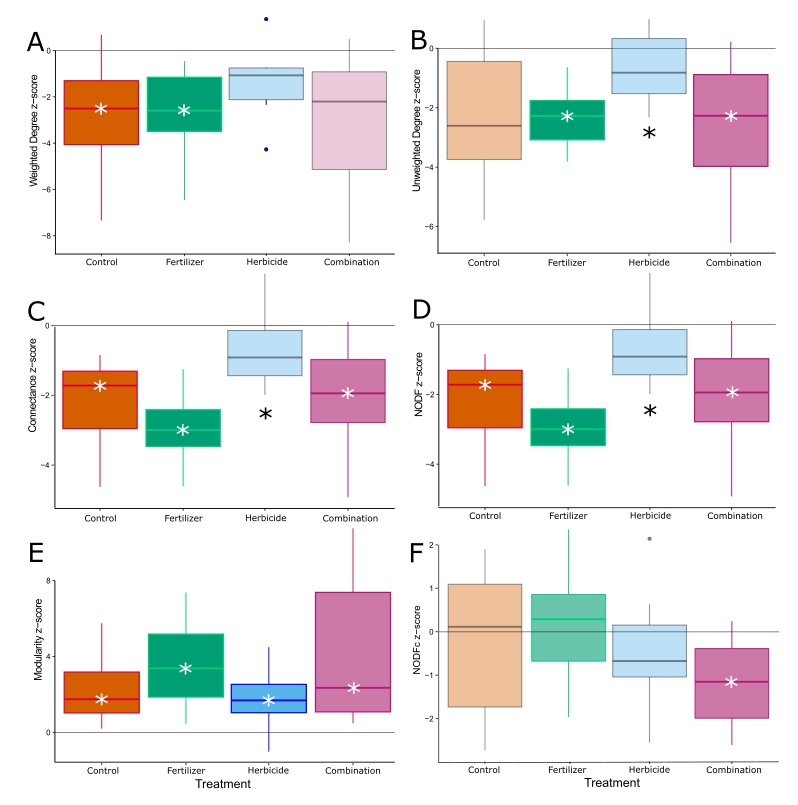


**Fig. S7.** Z-scores for (A) weighted degree, (B) unweighted degree, (C) connectance, (D) NODF, (E) modularity and (F) NODFc of the treatment networks from the study relative to null models (generated by r2dtable). We tested for significant differences between the agrochemical treatments and the control using GLMMs, and treatments that are significantly different than the control are indicated with a black asterisk. We also indicated the treatments that differed significantly from the null expectation with a white asterisk on the median line.

**Figure S8**


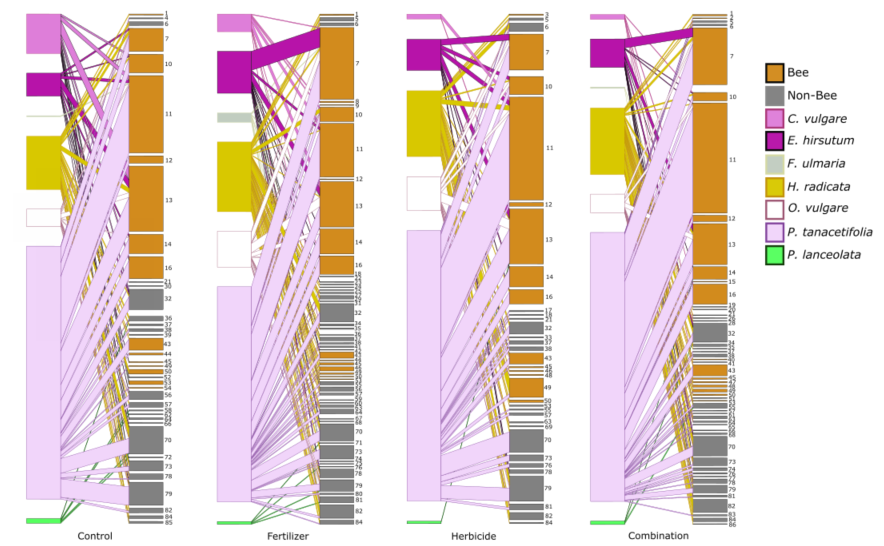


**Fig. S8**. Visualization of bipartite networks in the four experimental treatments, with plant species on the left side and insect species on the right side. These network images are generated from data aggregated across sites at the treatment level (i.e. all fertilized plots are pooled, etc.). *P. lanceolata*: green, *P. tanacetifolia*: light pink, *O. vulgare*: white, *H. radicata*: yellow, *F. ulmaria*: grey, *E. hirsutum*: purple, *C. vulgare*: dark pink, Non-bees: grey, Bees: orange. See Table S2 for species codes used in the figure.
