# Supplementary material for: Manipulating network connectance by altering plant attractiveness
Source: PeerJ. 2023 Nov 9;11:e16319. doi: 10.7717/peerj.16319 (PMC10640842; doi:10.7717/peerj.16319)
Supplement: Supplemental Information 4 — Map downloaded (edited for clarity) from the Geological Survey Ireland, Department of Communications, Climate Action & Environment, using the Teagasc soils layer (https://dcenr.maps.arcgis.com/apps/MapSeries/index.html, accessed 19/06/2019). Figure contains Irish Public Sector Data (Geological Survey) licensed under a Creative Commons Attribution 4.0 International (CC BY 4.0) license. All research plots were located on made ground. Filled squares represent sites established in 2018, and open squares represent sites established in 2017. This figure is modified from Russo et al. (2020). [file peerj-11-16319-s004.pdf]

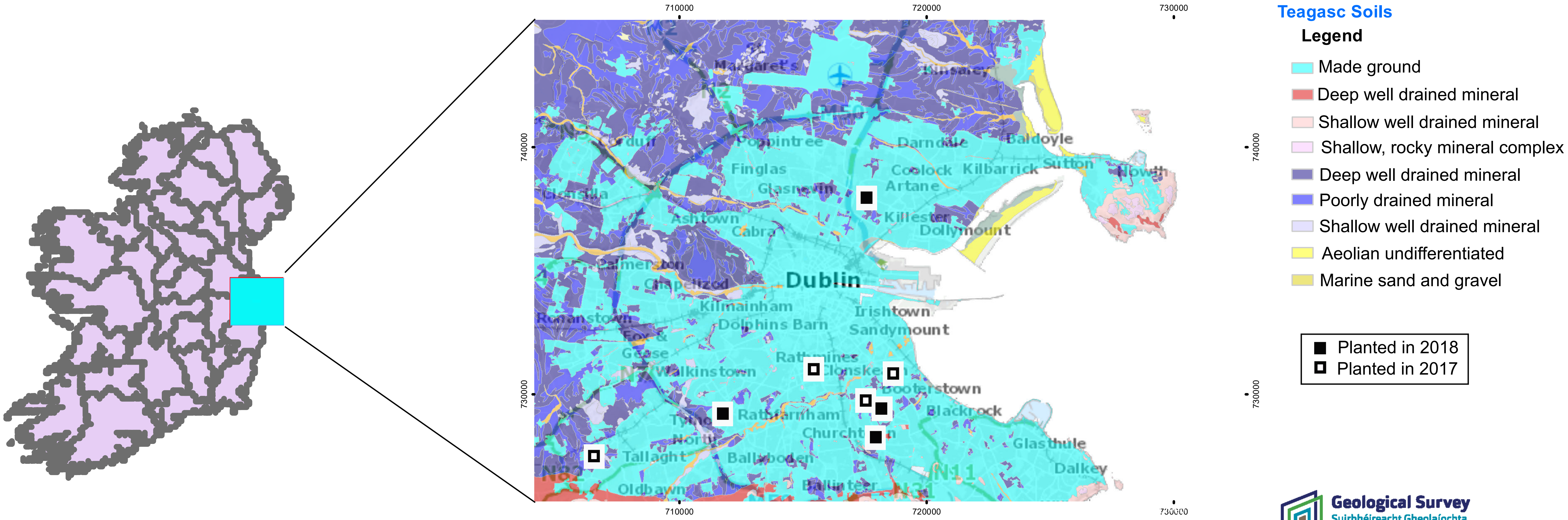

Scale: 1:200,000

Geological Survey Ireland

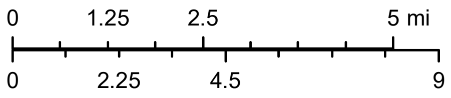

This map and its data may not be used or reproduced for commercial purposes without the prior written permission of Copyright holders.
This map is a user generated static output from an Internet mapping site and is for general reference only.
Data layers that appear on this map may or may not be accurate, current, or otherwise reliable.

Geological Survey
Suirbhéireacht Gheolaíochta
Ireland | Éireann

Ordnance Survey Ireland Licence No. EN 0047219
© Ordnance Survey Ireland/Government of Ireland
© Geological Survey Ireland/Government of Ireland
