# Supplementary figures and images for: Manipulating network connectance by altering plant attractiveness
Source: PeerJ. 2023 Nov 9;11:e16319. doi: 10.7717/peerj.16319 (PMC10640842; doi:10.7717/peerj.16319)

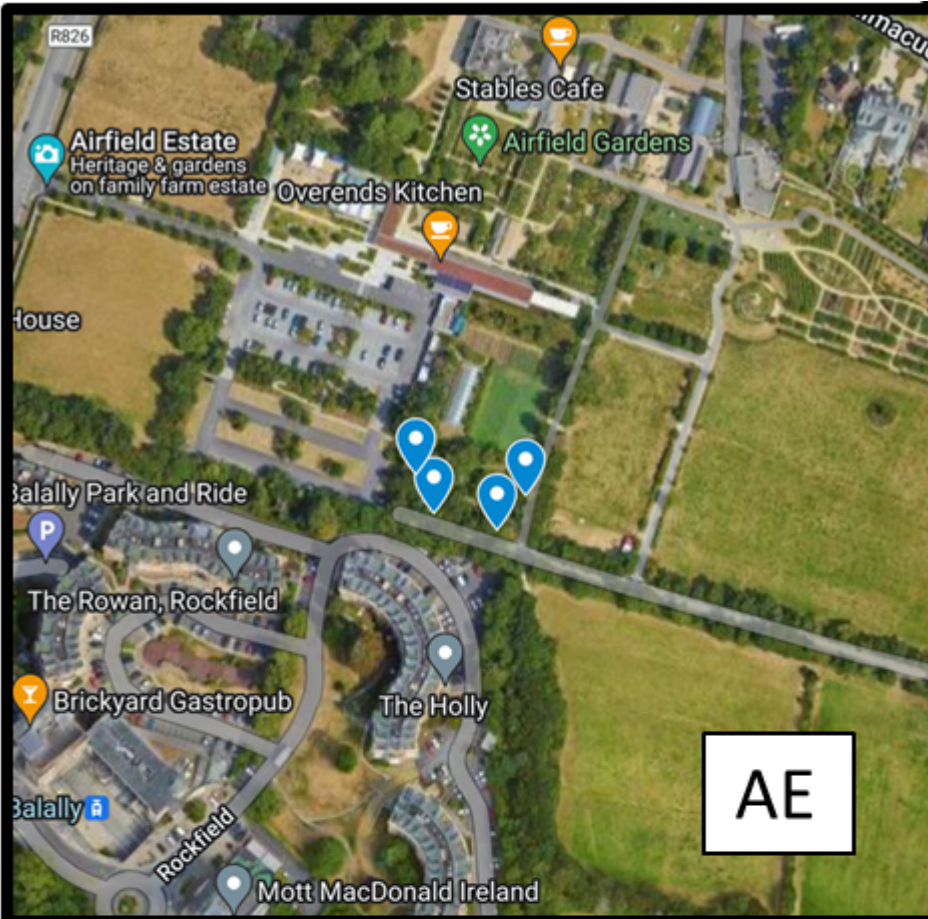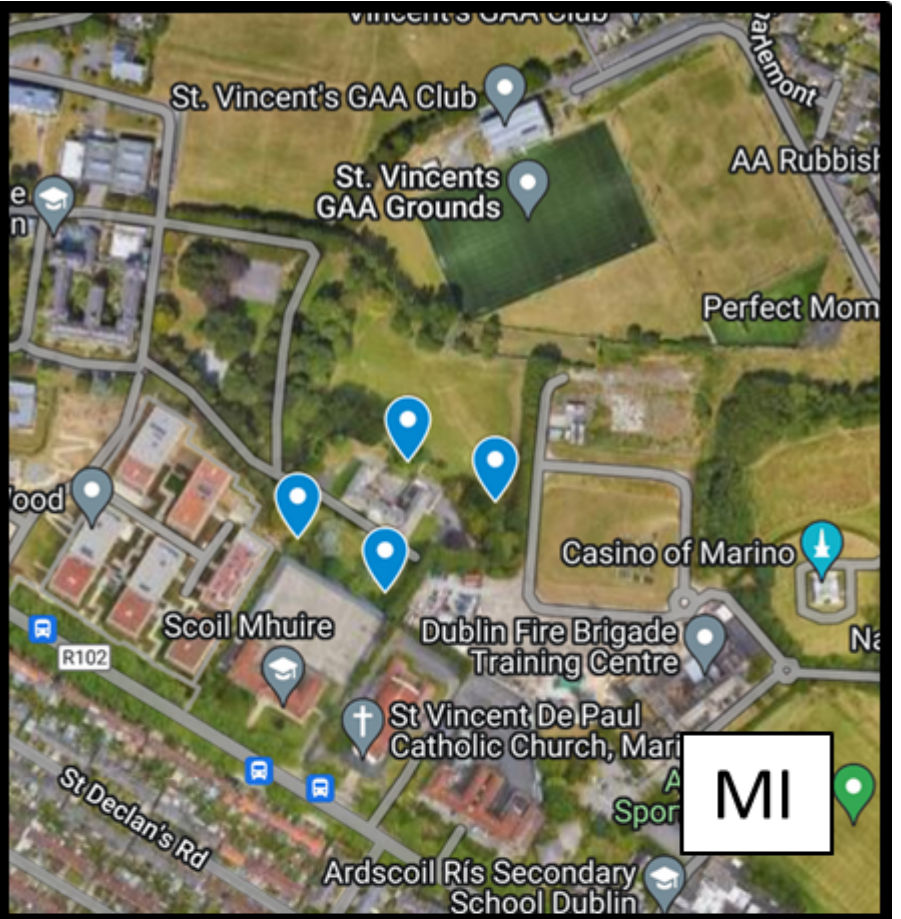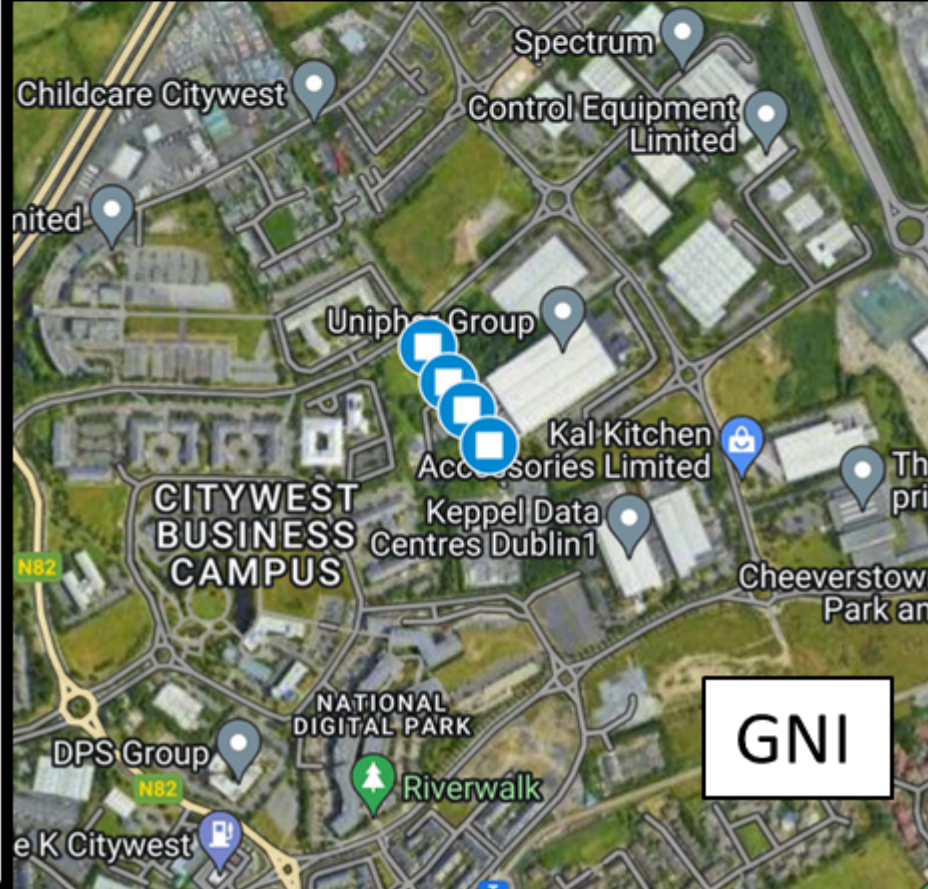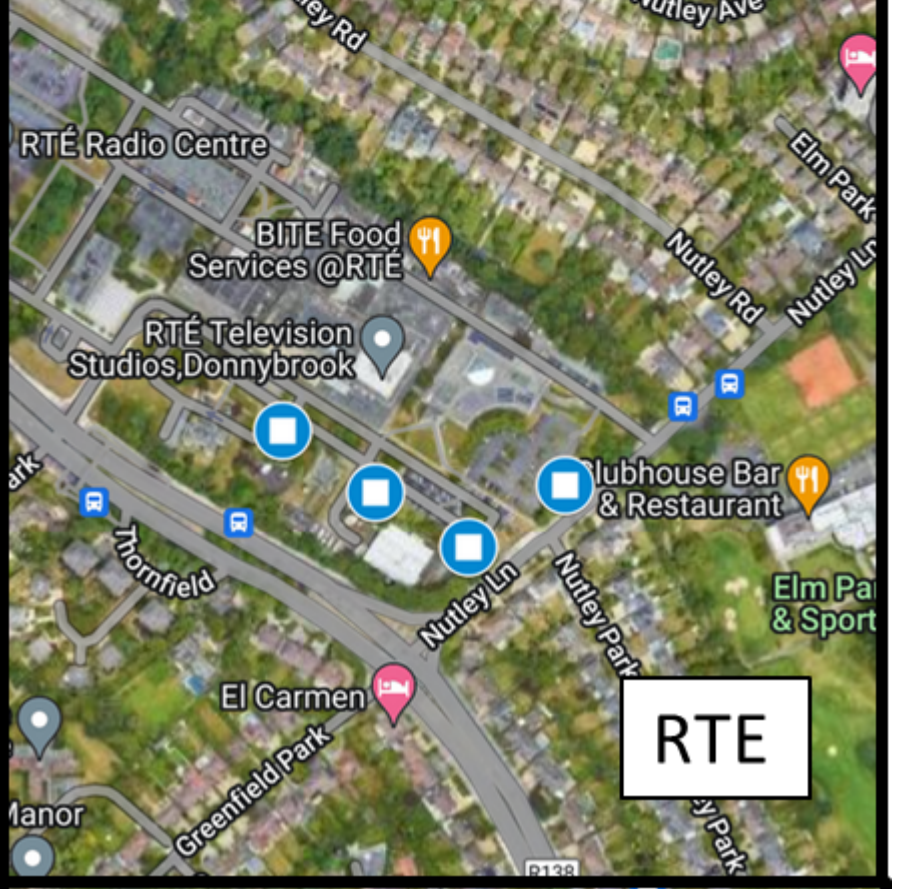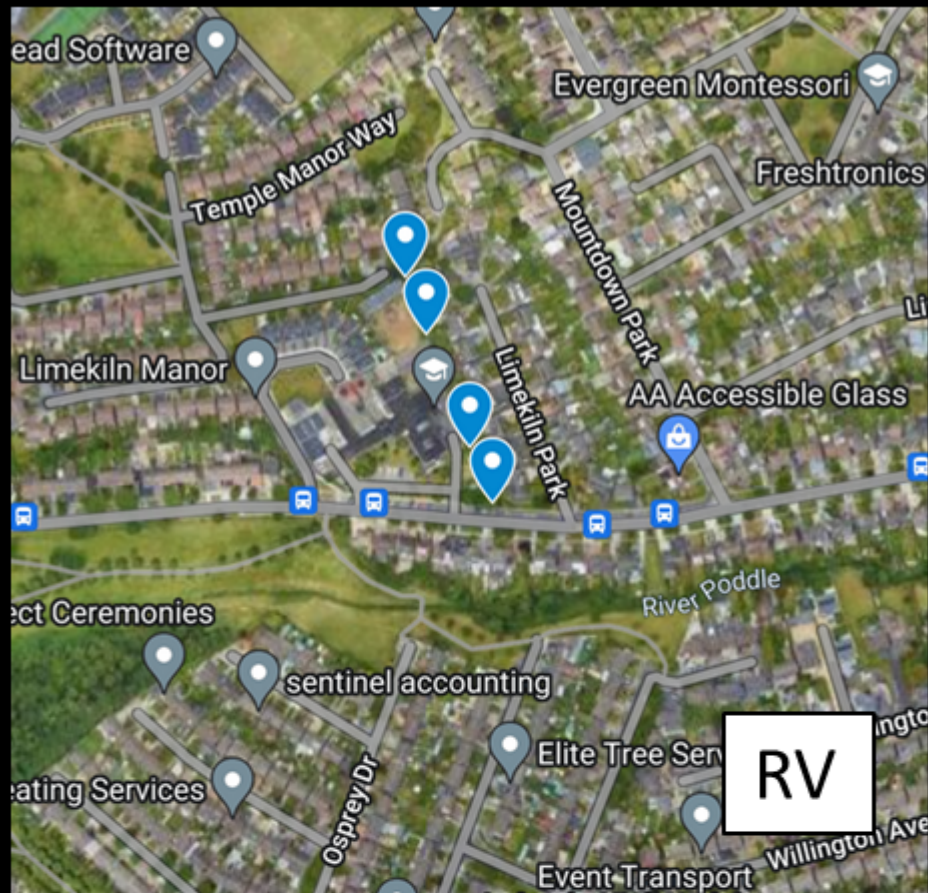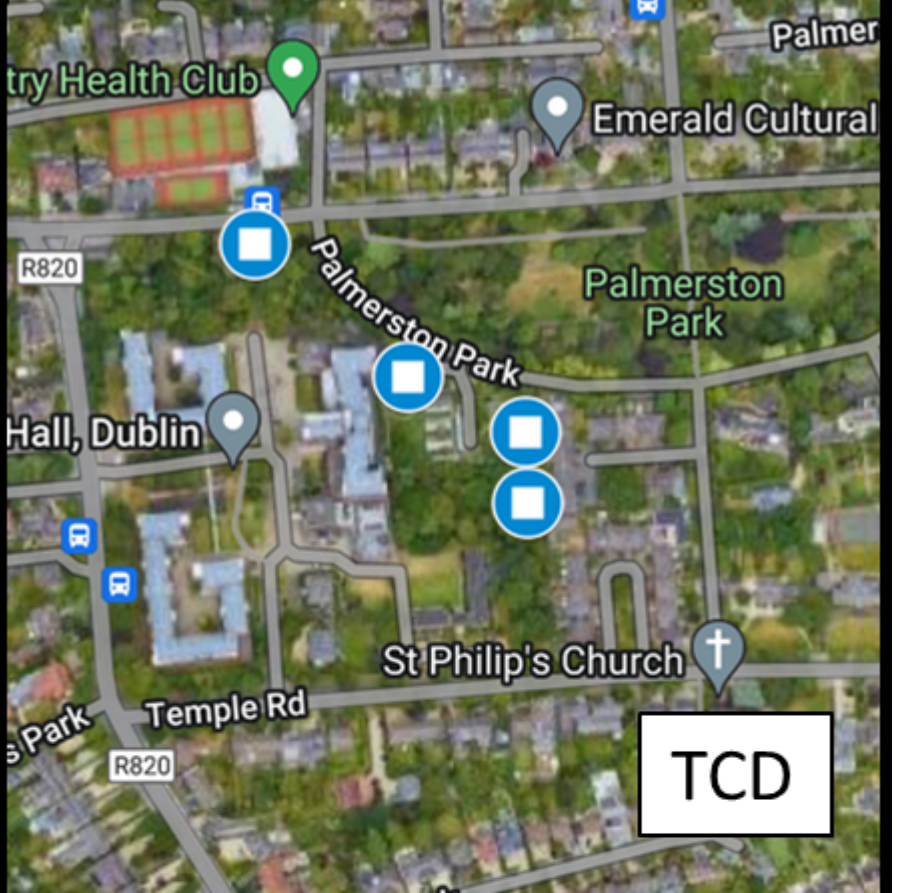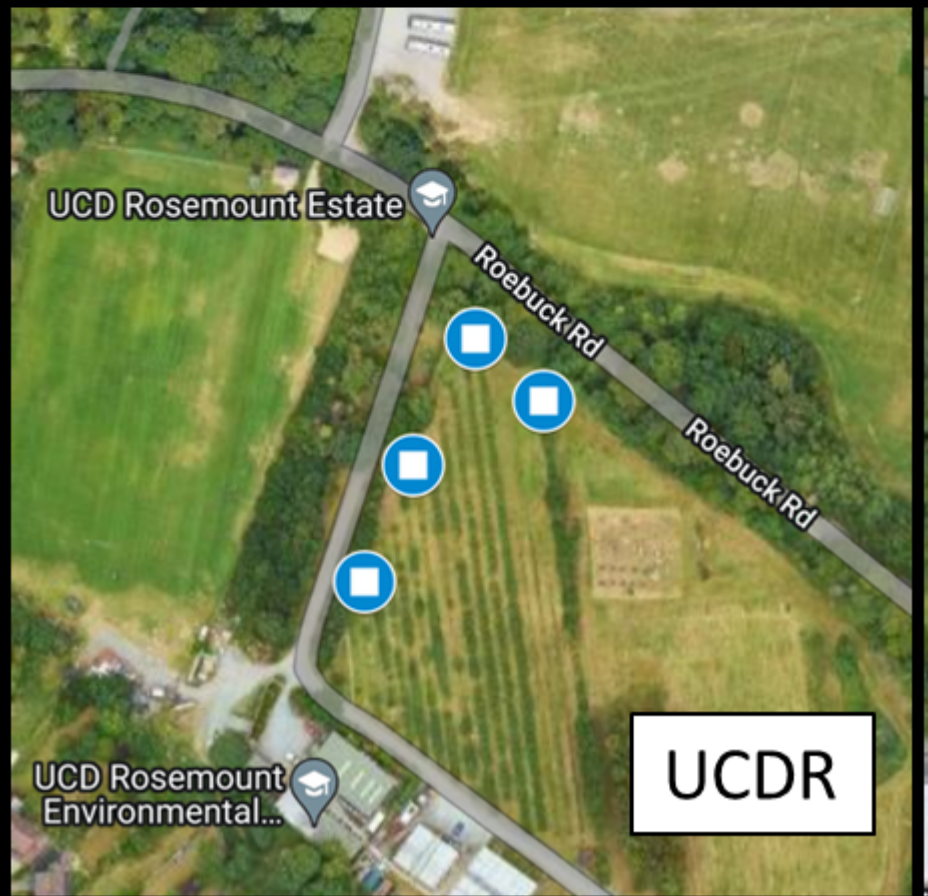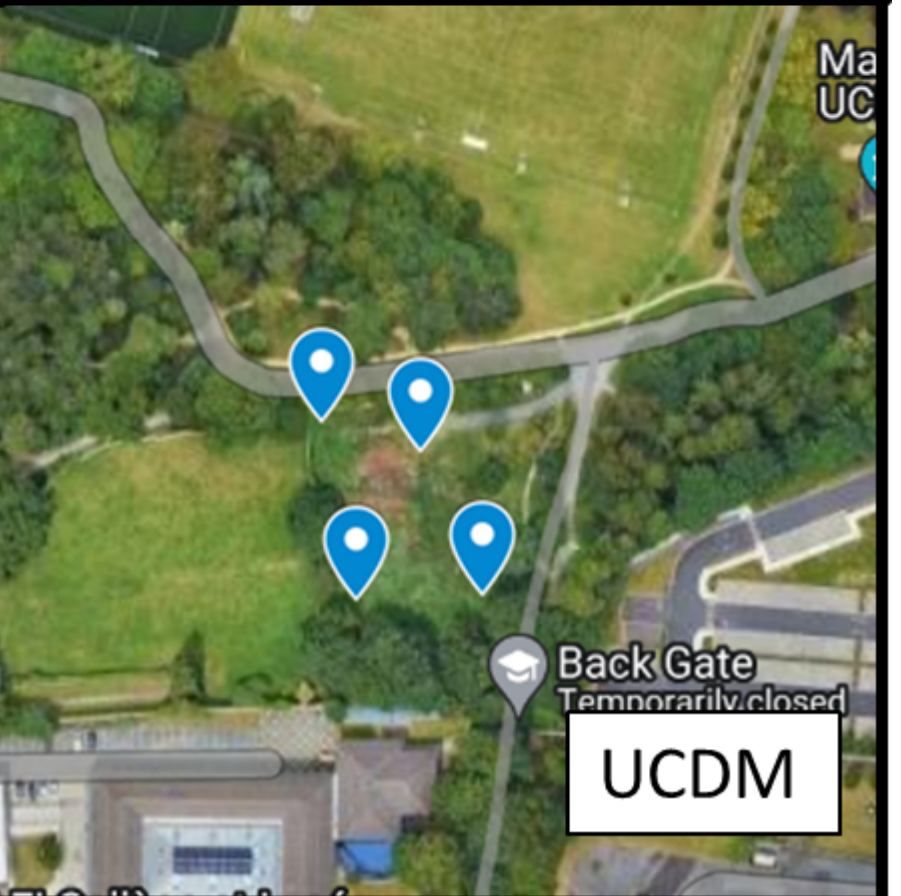

Supplement: Supplemental Information 5 — Plots established in 2017 are indicated by circles, while plots established in 2018 are marked by pins. [file peerj-11-16319-s005.pdf]

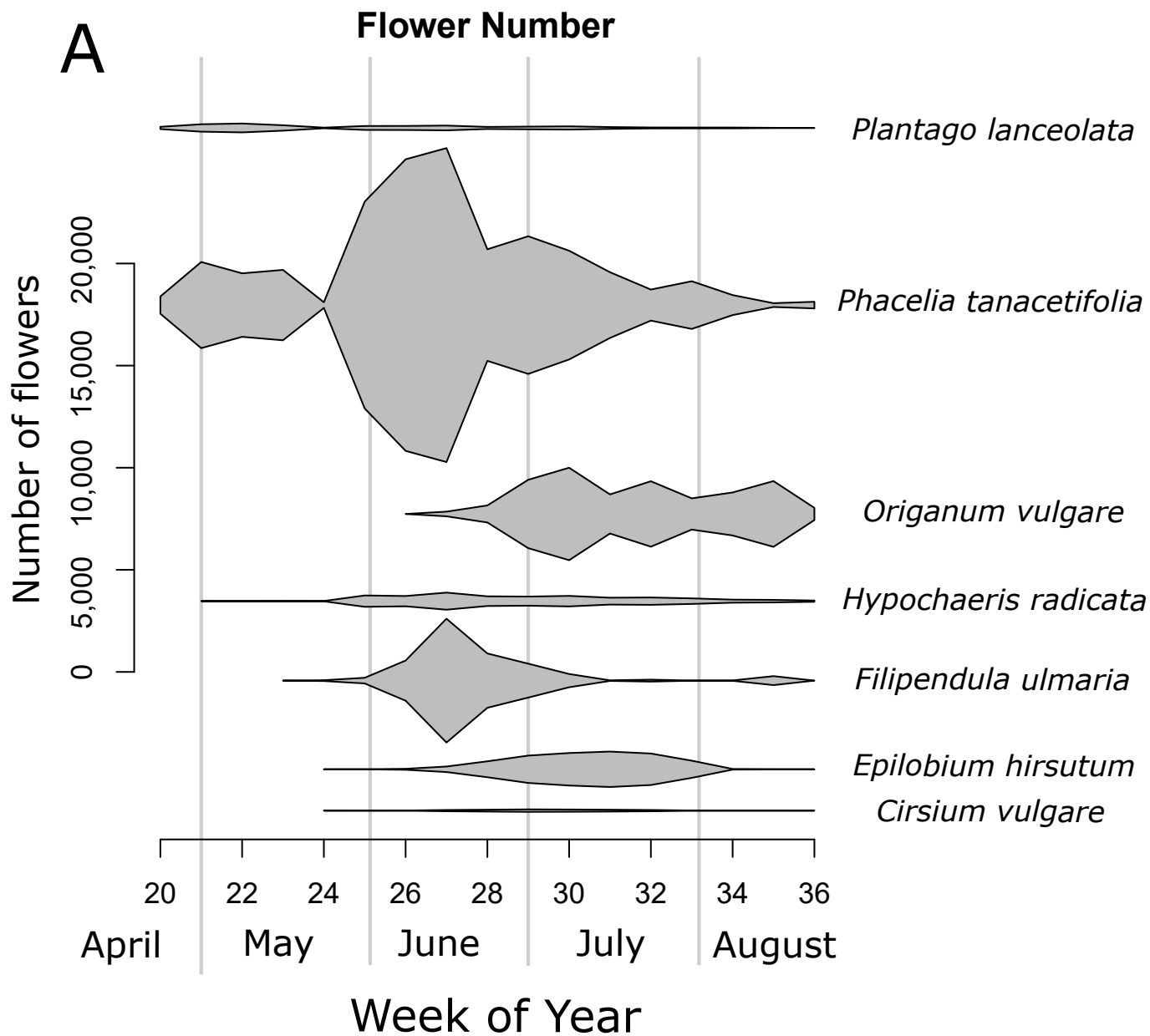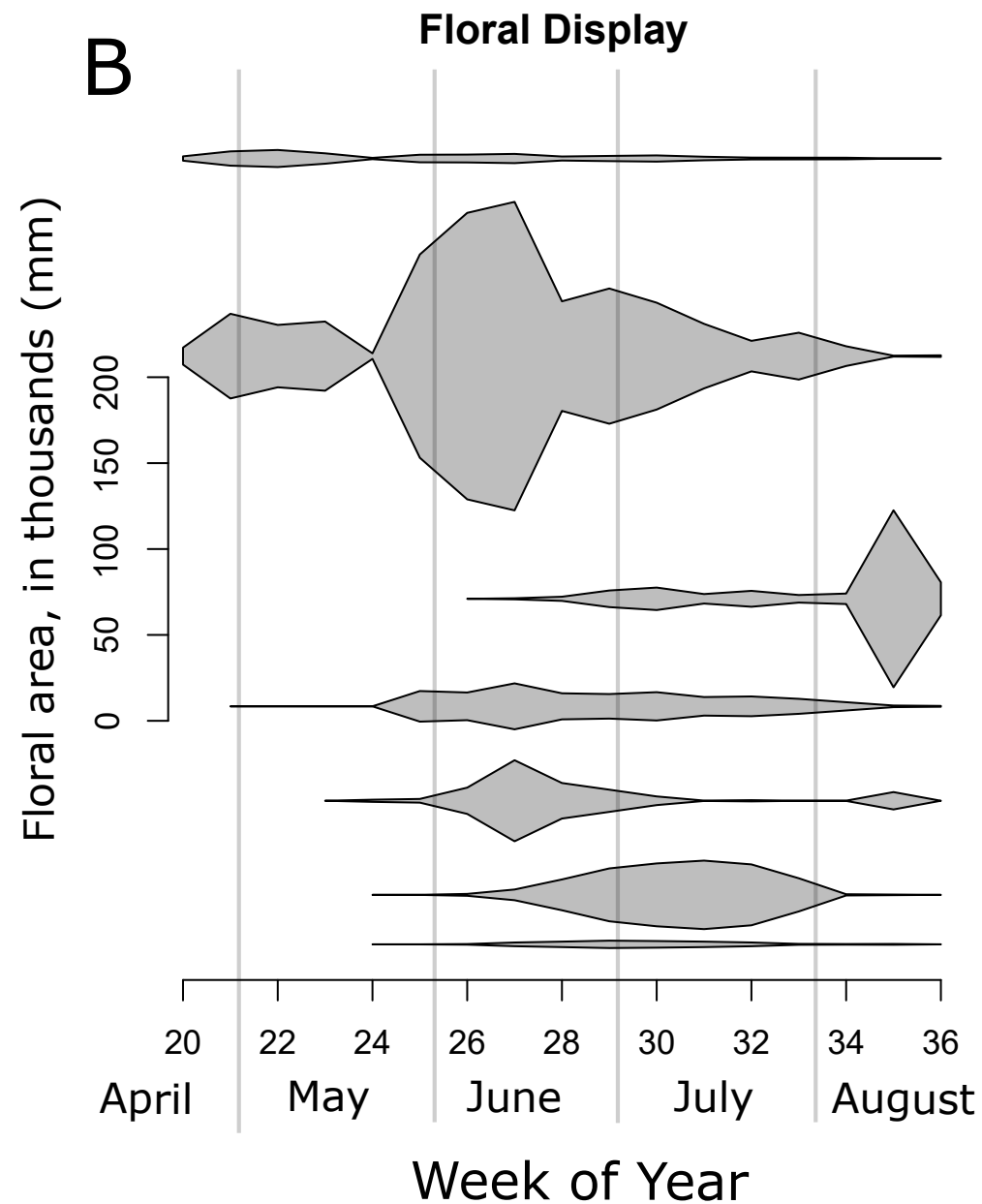

Supplement: Supplemental Information 6 — The first two plant species began to bloom in late April and continued to bloom through August for both years. Bloom in the seven species overlapped for most (10 out of 16 weeks) of the field season. [file peerj-11-16319-s006.pdf]

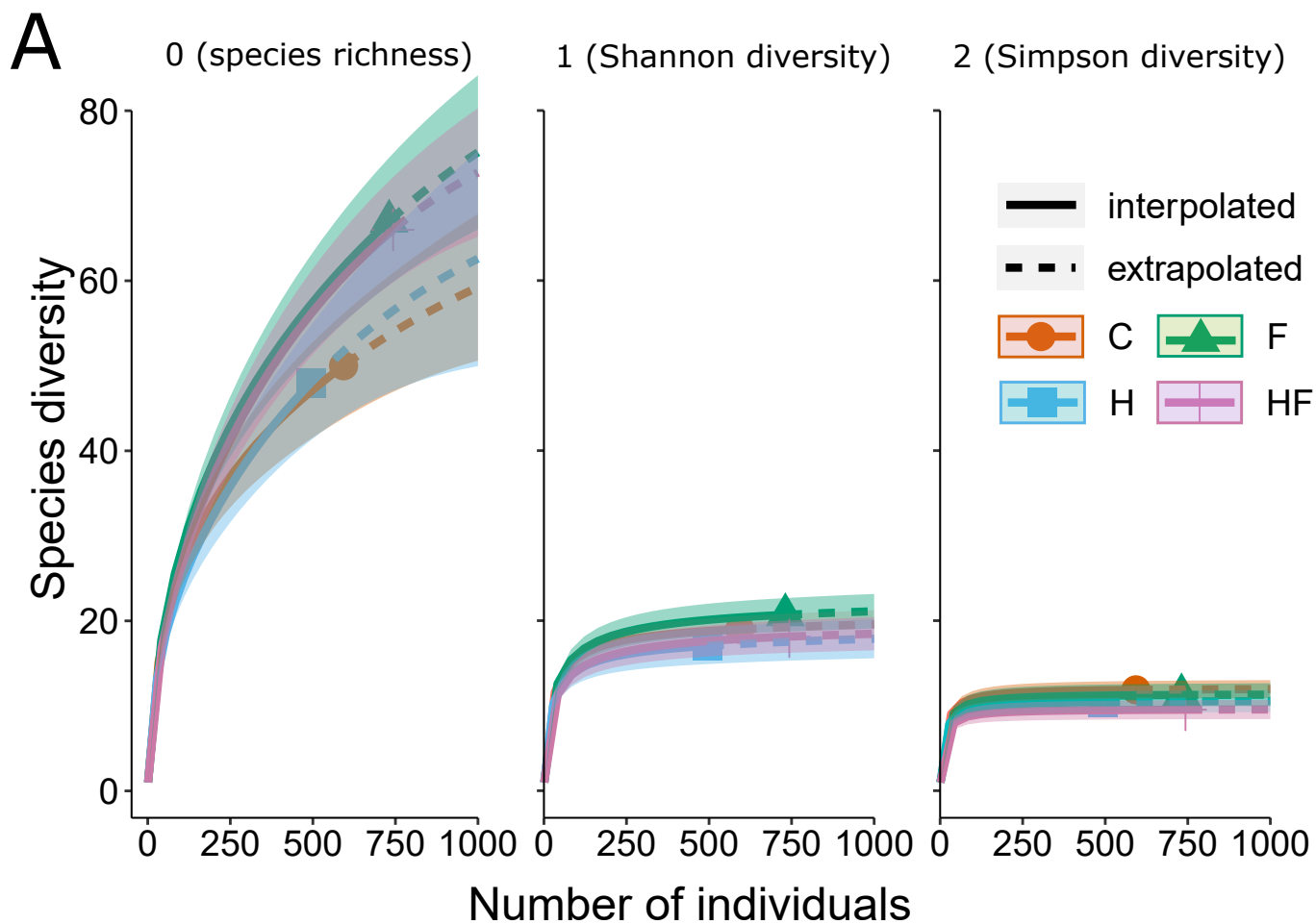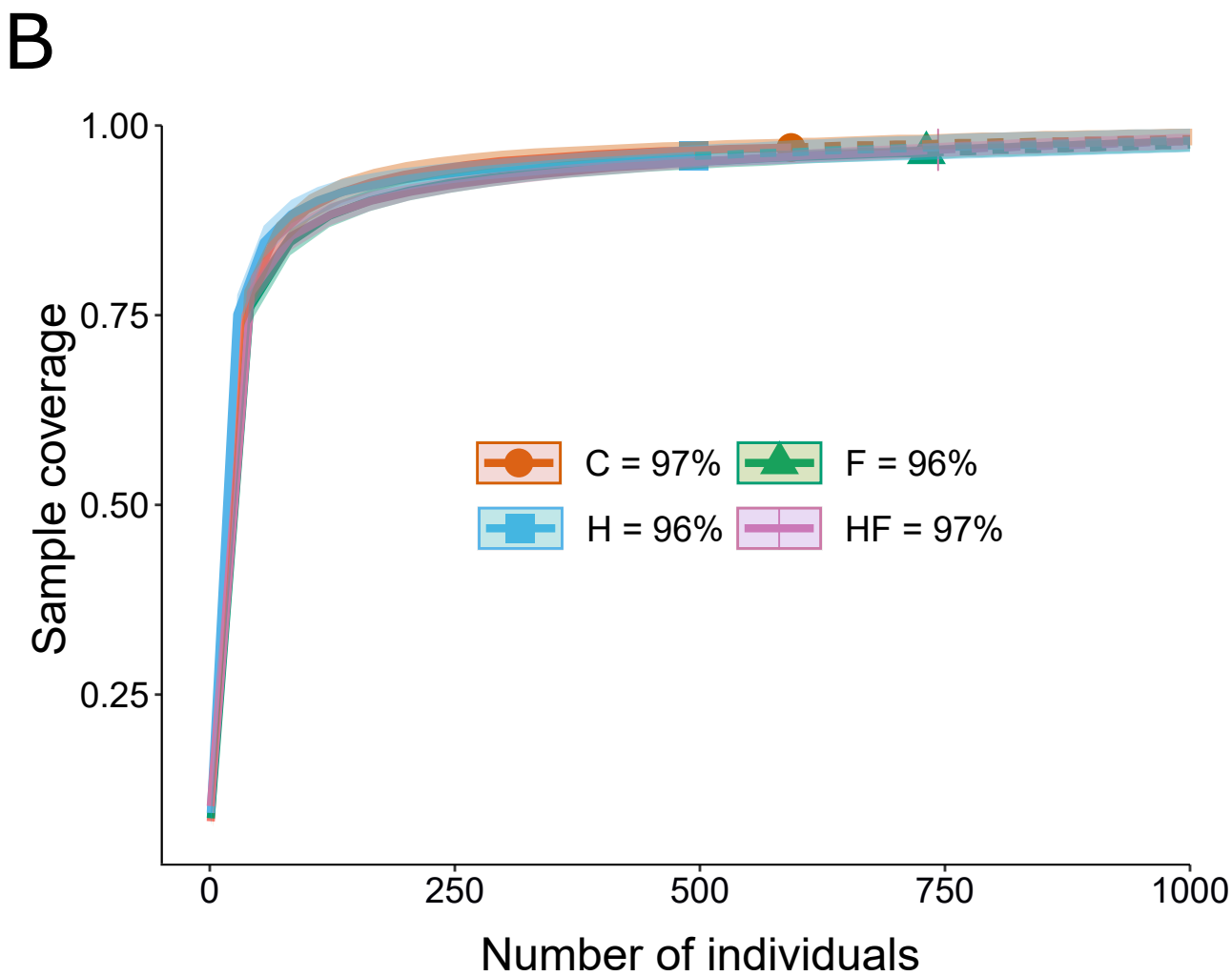

Supplement: Supplemental Information 7 — (A) shows the three Hill numbers among the four experimental treatments (C = control, F = fertilizer, H = herbicide, HF = combination). The shaded area around the lines indicates the 95% confidence interval of the expected diversity values of the four treatments. (B) shows the sample coverage for the four experimental treatments, including the percent of estimated diversity captured. In all treatments, we collect either 96 or 97% of the expected insect species richness. [file peerj-11-16319-s007.pdf]

**Correlation plot**

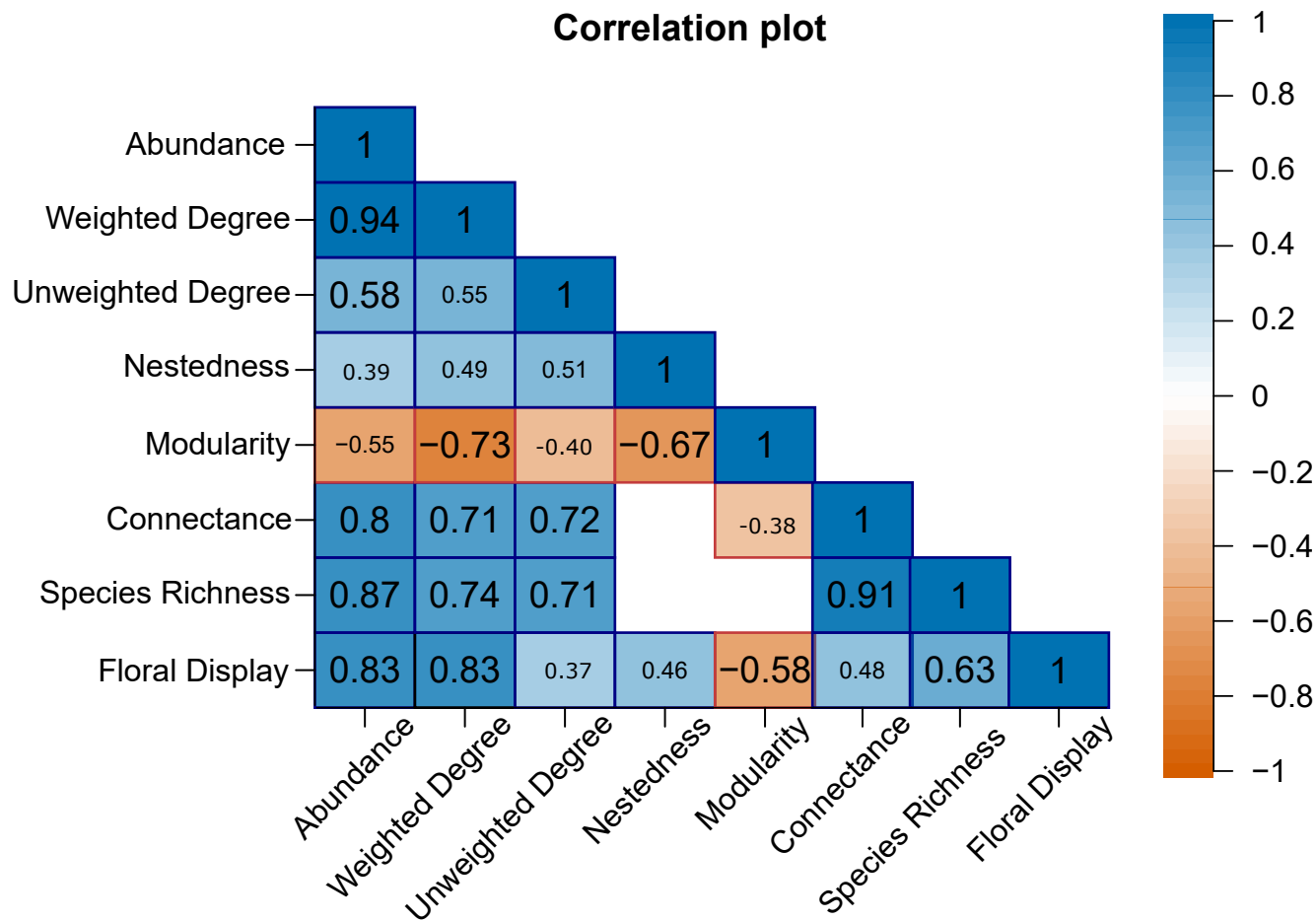

Supplement: Supplemental Information 8 — Numbers represent significant (P < 0.05) Pearson correlation coefficients. Larger font size indicates smaller P values. Negative correlations are indicated in red, while positive correlations are indicated in blue. Darker colors indicate correlation coefficients of greater magnitude. Associations in white with no numbers are non-significant (P > 0.05). [file peerj-11-16319-s008.pdf]

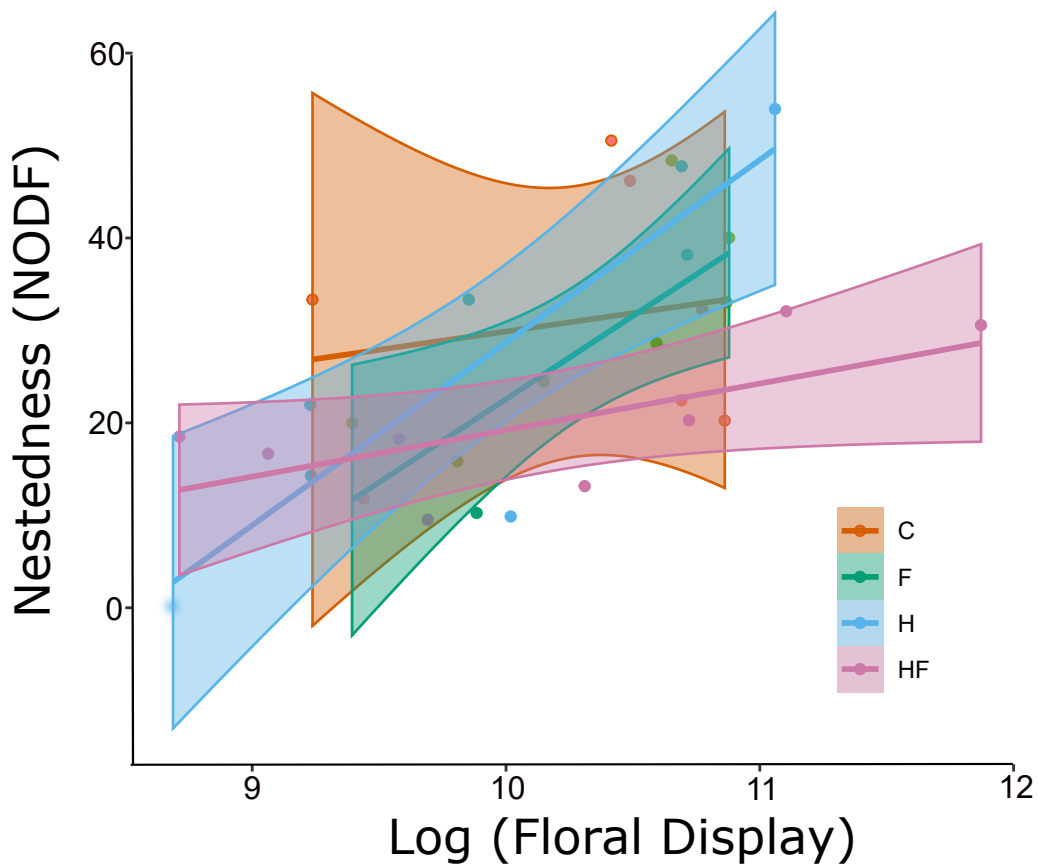

Supplement: Supplemental Information 9 — The colors indicate experimental treatment: red = control, green = fertilizer, blue = herbicide, purple = combination. There is a significant interaction between the size of the floral display and experimental treatment for network nestedness. [file peerj-11-16319-s009.pdf]

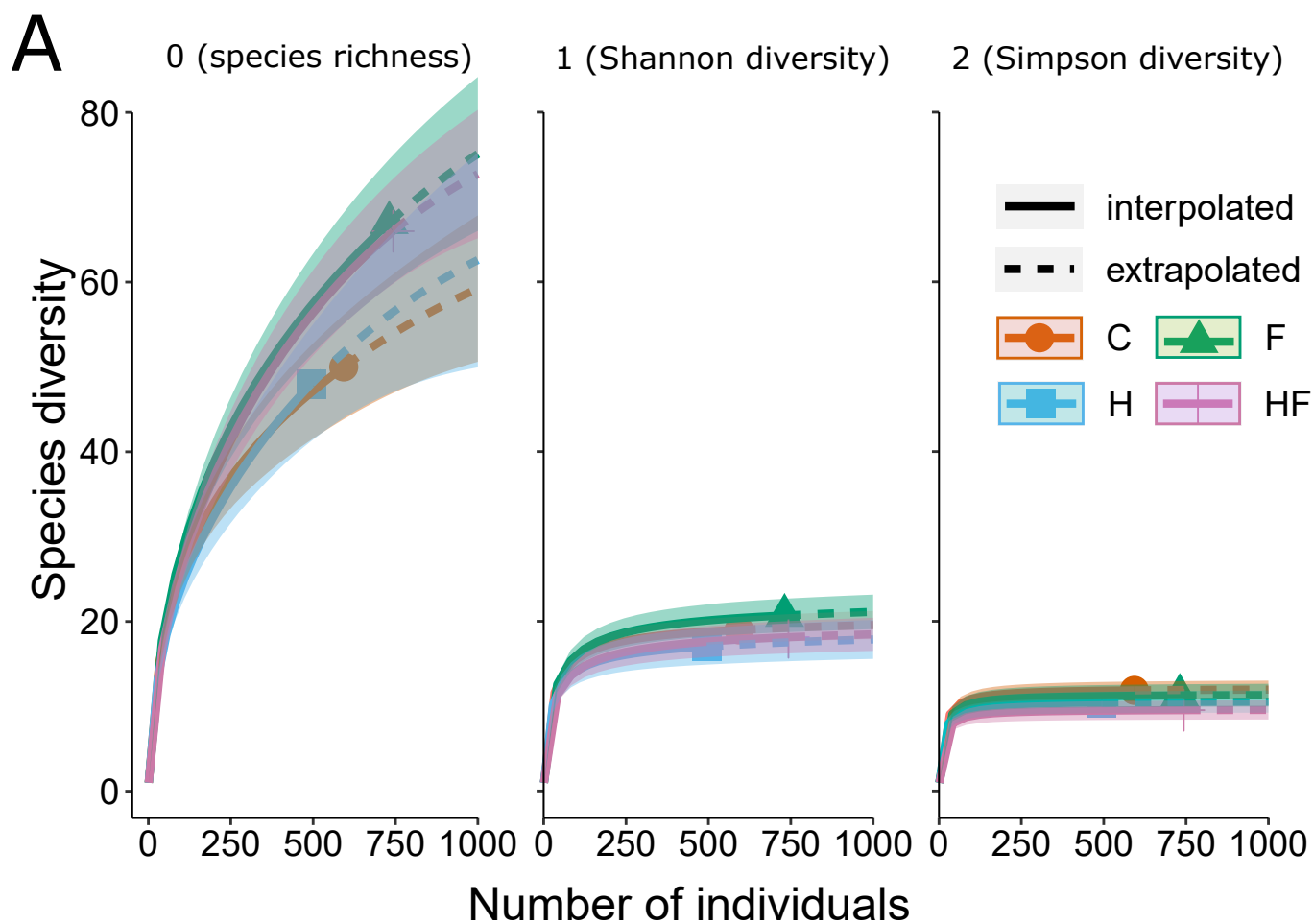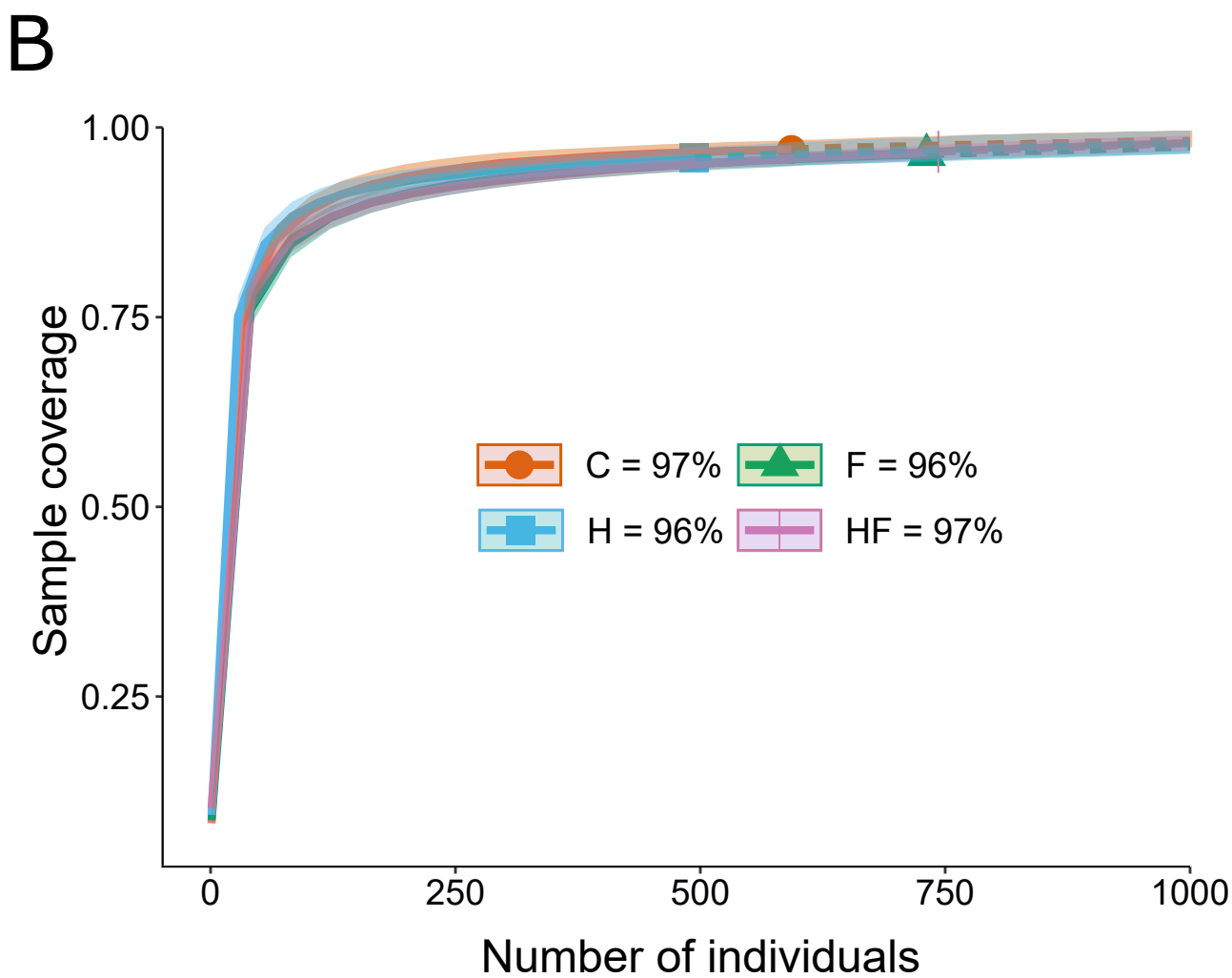

Supplement: Supplemental Information 10 — We tested for significant differences between the agrochemical treatments and the control using GLMMs, and treatments that are significantly different than the control are indicated with a black asterisk. We also indicated the treatments that differed significantly from the null expectation with a white asterisk on the median line. [file peerj-11-16319-s010.pdf]

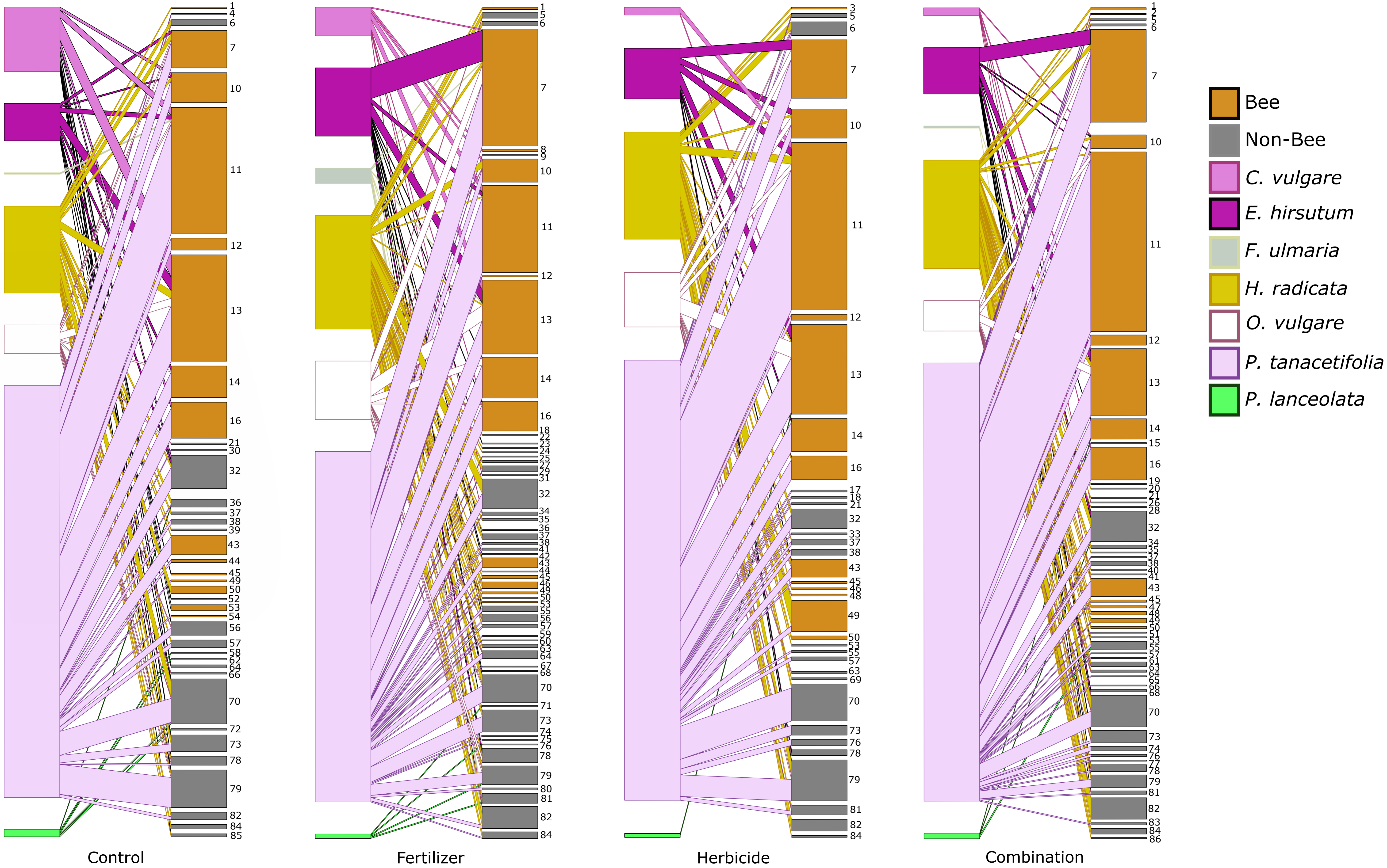

Supplement: Supplemental Information 11 — These network images are generated from data aggregated across sites at the treatment level (i.e., all fertilized plots are pooled, etc.,). P. lanceolata: green, P. tanacetifolia: light pink, O. vulgare: white, H. radicata: yellow, F. ulmaria: grey, E. hirsutum: purple, C. vulgare: dark pink, Non-bees: grey, Bees: orange. See Table S2 for species codes used in the figure. [file peerj-11-16319-s011.pdf]
